# Attracting states in frontal cortex networks associated with working memory and decision making

**DOI:** 10.1186/1471-2202-12-S1-P82

**Published:** 2011-07-18

**Authors:** Emili Balaguer-Ballester, Christopher C Lapish, Jeremy K Seamans, Daniel Durstewitz

**Affiliations:** 1 Bernstein-Center for Computational Neuroscience Heidelberg-Mannheim and Central Institute of Mental Health, Heidelberg University, J5, Mannheim, D-68159, Germany; 2 Department of Psychology, Indiana University Purdue University, Indianapolis, USA; 3 Brain Research Center & Department of Psychiatry, University of British Columbia, Vancouver, Canada

## 

A frequent hypothesis in theoretical neuroscience is that cognitive entities are represented and processed by attracting states of the underlying neural system [1]. For instance, different attractor-like states may represent different spatial locations or cognitive entities, and transitions between these attracting sets could be associated with the recall of a memory sequence or the execution of a motor plan. Attractor states underlying cognition were previously proposed in the context of working memory [1] and decision making tasks. However, although theoretically suggested, experimental evidence is still sparse for the hypothesis that higher cognitive processes proceed by moving between attracting states in higher cortical areas.

Using state space reconstruction theorems [3] and statistical learning techniques, we were able to reveal dynamical properties, not easily accessible in previous studies, of anterior cingulate cortex (ACC) multiple single-unit activity (MSUA) during a cognitive task. The approach worked by constructing high-dimensional state spaces from delays of the original single-unit instantaneous firing-rates and all possible products (multinomials) among them up to some specific order. The dynamics within these sparse and high-dimensional spaces of neural activity interactions were then statistically accessed using strongly regularized kernel methods [4,5].

Results showed cognitive-epoch-specific neural ensemble states (dependent on behavioral performance [3]) in ACC while the rats performed an ecologically valid eight-arm radial arm-maze task. More interestingly, these cognitively defined ensemble states showed some hallmarks of attracting behavior which became apparent in high-dimensional expansions of the MSU spaces due to a proper unfolding of the neural activity flow. It turned out that optimal unfolding of neural trajectories was achieved in an embedding space characterized by a specific maximum order of neural interactions, common across different animals. In further analyses, ensemble states as a function of the animal’s spatial position i.e. the arm visited were analyzed. From these analyses the *intrinsic* dimensionality which is relevant to the animal’s arm choices could be computed [4]. Results showed that cognitively relevant network states were restricted to a low-dimensional nonlinear manifold within the high-dimensional space. Finally, preliminary analyses indicate that it might be possible to directly infer from this reduced space an idealized neural ensemble model with predictive properties across future trials with respect to the animal’s arm choices. An analysis of such a model would permit a full characterization of the putative neural attractors underlying working memory. To summarize, results suggest that ACC networks may process different subcomponents of higher cognitive tasks by transitioning between different attracting states. Moreover it may be possible to use these analyses to characterize the dynamical states underlying the animal’s choices without further assumptions about the biophysical variables.
